# Vision-Based Sensor for Early Detection of Periodical Defects in Web Materials

**DOI:** 10.3390/s120810788

**Published:** 2012-08-06

**Authors:** Francisco G. Bulnes, Rubén Usamentiaga, Daniel F. García, Julio Molleda

**Affiliations:** Department of Computer Science, University of Oviedo, Campus de Viesques, Gijón 33204, Spain; E-Mails: rusamentiaga@uniovi.es (R.U.); dfgarcia@uniovi.es (D.F.G.); jmolleda@uniovi.es (J.M.)

**Keywords:** vision sensors, intelligent systems, automated defect detection, pattern recognition

## Abstract

During the production of web materials such as plastic, textiles or metal, where there are rolls involved in the production process, periodically generated defects may occur. If one of these rolls has some kind of flaw, it can generate a defect on the material surface each time it completes a full turn. This can cause the generation of a large number of surface defects, greatly degrading the product quality. For this reason, it is necessary to have a system that can detect these situations as soon as possible. This paper presents a vision-based sensor for the early detection of this kind of defects. It can be adapted to be used in the inspection of any web material, even when the input data are very noisy. To assess its performance, the sensor system was used to detect periodical defects in hot steel strips. A total of 36 strips produced in ArcelorMittal Avilés factory were used for this purpose, 18 to determine the optimal configuration of the proposed sensor using a full-factorial experimental design and the other 18 to verify the validity of the results. Next, they were compared with those provided by a commercial system used worldwide, showing a clear improvement.

## 1. Introduction

**Figure 1.**
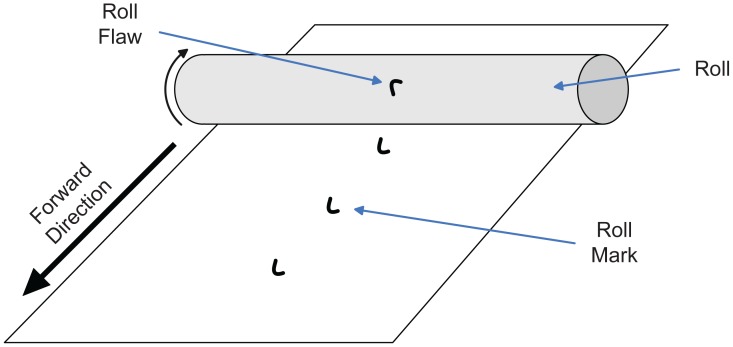
Defect generation due to a defective roll.

Quality control in industrial environments is an essential aspect, both to improve product quality and to reduce costs caused by discarded defective products. The current trend is to replace human experts for automated systems, since they are cheaper, take less time and can work even in the most dangerous environments [1]. This study focuses on quality control during production of web materials using a computer vision system. Such materials can be defined as those which are produced by continuous rolling processes, such as paper, plastic, fabric or metal. The rolls that are in direct contact with the web material to be produced can be the source of serious defects. When their surfaces have some kind of flaw, they can generate a surface defect on the rolled product each time they complete a full turn, generating a periodical pattern as shown in Figure 1. In these cases, this situation is repeated until the defective roll is replaced. For this reason, it is very important to make early detection of such defects during the production of web materials. The sooner this situation is detected, the sooner the defective roll may be replaced, greatly reducing the productivity loss.

**Figure 2.**
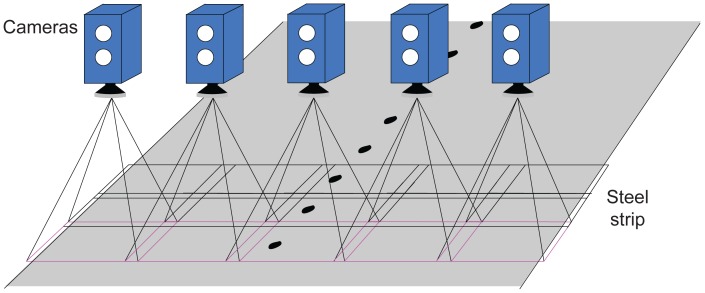
Cameras used in top surface inspection.

There are several studies about the development of vision-based sensor systems for industrial use, such as inspection tasks [2] or defect detection tasks in many different fields, like satin glass [3], welds [4], bearings [5] or ship hulls [6]. Depending on each case, the design of the vision system (cameras, lenses, lighting, *etc.*) is different. It is critical to build the most suitable vision system in each case in order to obtain satisfactory results [7]. In the particular case studied here, the sensing tasks are carried out using several cameras, which take images of both surfaces of the material to be inspected. The areas inspected by the cameras are overlapped in order to ensure complete coverage, as shown in Figure 2. These images are used by a software system to perform other typical tasks of these systems, namely fault detection, characterization of defects, feature extraction and classification [8]. Using an inspection system that performs these tasks, the technicians responsible for quality control can obtain information about the position and class (or type) of each defect present in the produced material. Some of these systems are able to determine whether a surface defect is a roll mark or not, based on its features. Thus, a technician can find out that at least one roll is defective. In production lines in which only one roll can generate defects, this information may be sufficient. However, in more complicated cases, where several rolls can be the source of such defects, more information about the periodical defects generated is required to determine which roll must be replaced.

In the most adverse situations, there may be several defective rolls generating defects simultaneously. To handle these situations, it is desirable to cluster the defects that have been generated by the same flaw in the same roll. The characteristics of each cluster of defects can be used to identify the defective roll. The separation between defects of the same cluster may be used for this. Normally this separation coincides with the perimeter of the roll that generated the periodical defect (if the radius of each roll involved in the production process is different, it would identify the defective roll unequivocally).

An additional phase for the design of a sensor capable of detecting periodical defects is proposed: the clustering phase. Its objective is the detection of surface defects generated periodically based on data provided by traditional inspection systems: position, type and features of each surface defect. This latter phase is performed by a software module that executes a clustering method. The main problem to develop this method is that the input data may be very different depending on the web material being inspected. As the inspection system and the production process may differ, the characteristics of the input data can vary greatly. The number of surface defects per square meter and the types in which they can be classified may be very different. In addition, inspection systems always add error to their measurements, which are different depending on the system used [9]. Therefore, the clustering method must take this into account. It should be able to be adapted to work with input data with different noise levels. Furthermore, in order to calculate the results quickly, consumption of computational resources and memory must be low. Thus, early detection of periodical defects can be achieved even without using a powerful computer.

A method that can detect such defects quickly even in noisy environments is proposed. In order to assess the proper operation of the whole sensor, it has been used in a real situation: the detection of periodical defects in hot steel strips. In this case, there are several rolls which can generate periodical marks (in our example there are 7 rolls) and the input data are very noisy. For these reasons, each strip can have a very high number of surface defects, making the task of detecting periodical defects much more difficult.

Less evolved versions of this method have been published previously by the authors (Bulnes *et al.* [10], Bulnes *et al.* [11], Bulnes *et al.* [12]). With more features to define the periodical defects (Subsection 3.1), improvements in the clustering algorithm (Section 3.2) and more configuration parameters (Section 3.3), better outputs are achieved. In addition, all steps taken by the proposed method are explained in detail, in order that anyone can reproduce it.

This paper is organized as follows. In Section 2, we review previous work related to the detection of periodical defects in web materials. The proposed method to perform periodical defect detection is introduced in Section 3. Specifically, it describes the way the information is managed, the detection algorithm and its parametric optimization. Section 4 presents the utilization of the proposed method to a practical application: hot steel strips, in order to verify that it can be used in a real industrial environment. Finally, in Section 5, we summarize the main conclusions of the paper.

## 2. Related Work

The available literature about the detection of periodical defects is scarce. Although many of the commercial systems that are used to carry out inspection tasks of web materials are able to detect these defects, the information about this process is not public. However, there are some published studies where periodical defects are detected in different types of web materials, which are discussed below.

### 2.1. Paper

Paper is usually made using wood pulp. A typical paper machine consists of the wet end (or forming section), the press section and the drier [13]. In the wet end, the sheet of paper is formed by passing the mixture of pulp and water between two porous belts called forming fabrics. In the press section, most of the water is removed by applying pressure to the sheet using several rollers. Finally, any remaining moisture is evaporated using heated rolls in the drier. Commonly, a final stage is also used: the calender section, where the paper can obtain a smoother and more uniform surface by applying pressure to the already dry paper using rolls.

Although many rolls are involved in the paper making process, most of the periodical defects are produced in the press section, because a high pressure is applied when the paper is still in a delicate and deformable state [14]. The authors of the said paper were able to detect the periodical defects using two-dimensional fast Fourier transforms. However, several periodical and pseudo-periodical defects may appear in the wet end too. In [15,16], periodical defects generated in the wet end were detected using Fourier analysis, and periodical defects generated in the wet end and press section were also detected using the same technique by Chaplin [17].

### 2.2. Textiles

Depending on the type of fabric, the production process can vary greatly. Moreover, unlike what happens in the case of paper or in the case of hot metal, textiles are not in such a delicate state during the production process, which reduces the possibility of generation of periodical defects. During the production of textiles, inspection systems detect and classify surface defects [18], but rarely is the task of detecting periodical defects performed.

The first study that includes the detection of periodical defects was performed by Toba [19]. In this study, autocorrelation functions were used to inspect products and carry out the detection of periodical defects. This method was also used by Drean *et al.* [20] to detect periodic irregularities in textile surfaces. Furthermore, a variance analysis was also used. Another recent example of the defects that were made periodically is studied by Kianiha *et al.* [21], where fabric products are analyzed using image analysis methods.

### 2.3. Metal

During the production of metal products, such as rods, tubes or strips, the metal leaves the furnace at very high temperatures. While still hot, using a series of rolls, its desired shape is obtained. At this point the metal is very malleable, which favors the appearance of periodical defects due to flaws in any of the rolls. In these cases, the inspection task is usually performed using computer vision techniques, although it depends ultimately on the type of product to be inspected.

The metal product for which most studies of this kind have been made are steel or iron strips. In Duan *et al.* [22], a neural network is used to classify each surface defect within a strip depending on its characteristics. Since all defects generated periodically by defective rolls have similar characteristics, this classification can be used to warn about that situation. Similarly periodical defects are detected in [23]. However, Shigeno *et al.* [24] proposes a method to detect periodical defects based on the position of each surface defect, instead of using its characteristics. This method is similar to the one proposed in this paper, described in Section 3.

This type of detection is also performed during the production of elongated metal products such as tubes or wires. In Traxler *et al.* [25] a method for the detection of defective rolls in the production of seamless steel tubes is proposed. In the said study, the detection is performed taking into account the characteristics of each surface defect and also its position. Moreover, Park *et al.* [26] proposes two methods for detecting and identifying periodical defects in steel wire rods. Their detection is performed using a Discrete Wavelet Transform, and their identification is carried out using a Discrete Fourier Transform.

### 2.4. Other

Periodical defects can also occur in other types of web materials. For example, in Oerley *et al.* [27] periodical defect detection in plastic film material is performed using a correlation function.

In Caron *et al.* [28], the inspection of preweathered zinc is presented. The inspection system used in this case detects periodical defects within zinc strips which are submitted to a process (called preweathering) that gives the strip a slate gray color. During both the production process of the strip and preweathering, periodical defects may appear. In this study, the surface defects are detected using computer vision and classified based on their characteristics. The system only warns about defective rolls when there are defects which have been classified as “roll mark”.

## 3. Proposed Approach

The proposed approach will perform the detection of periodical defects using a method whose objective is to look for surface defects that meet certain conditions. In this section the main contributions of the paper are presented: the way to represent a periodical defect as a pattern defined by 9 features and the parameterizable backtracking based algorithm responsible for carrying out the detection and characterization of these defects.

**Figure 3.**
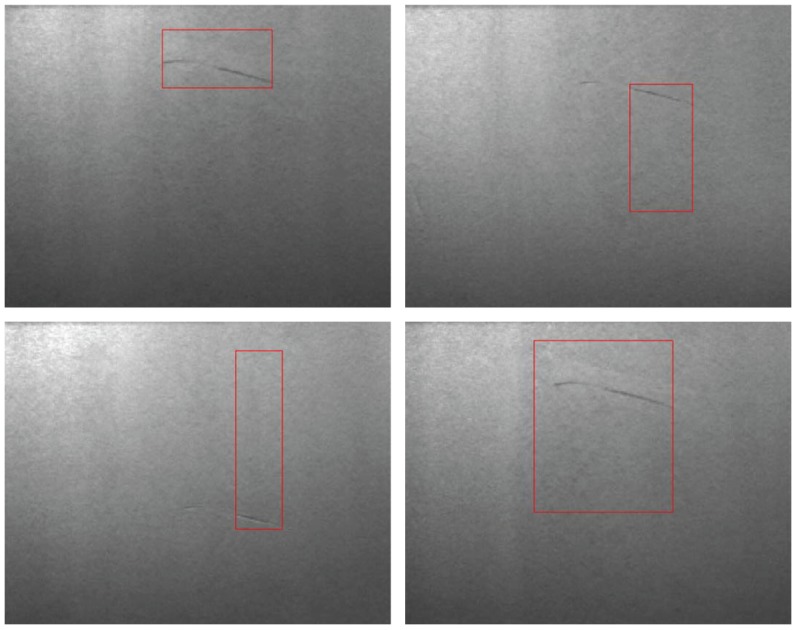
Inaccurate input data.

As mentioned above, the uncertainty introduced by the inspection system must be taken into account. Figure 3 shows four different individual defects generated by a defective roll during the production of a steel strip. They all have the same shape, but the dimensions estimated for each of them by the inspection system are very different. Consequently, the position assigned to each is not correct, since it is calculated as the central point of the square surrounding the defect. Although these cases are extreme, the inspection system always introduces an uncertainty in the characteristics of each individual defect (this error may be higher or lower depending on the web material inspected and the inspection system used). Since the type assigned to each surface defect depends on its characteristics, if they are not precise the assigned type may also be incorrect.

Before explaining its operation, it is necessary to establish a series of definitions to be used in the rest of the document.

**Figure 4.**
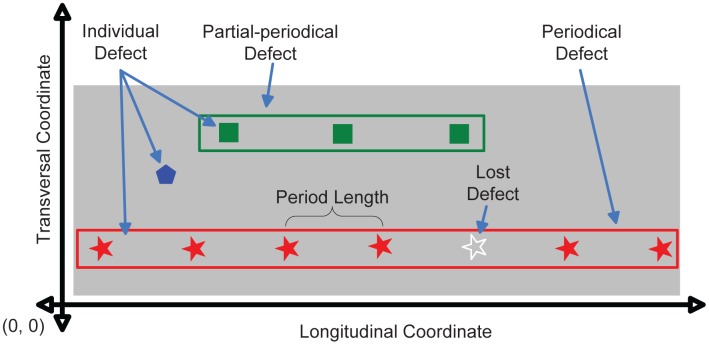
Defect definitions.

- **Individual Defect:** Each surface defect is called an individual defect. In Figure 4, each polygon represents a different individual defect. The relevant characteristics of each individual defect can be grouped into two categories: spatial and morphological. Spatial characteristics determine its position within the surface of the web material (both transversal and longitudinal coordinates). Morphological characteristics define its shape. These characteristics, provided by the inspection system, are its area (in pixels), its type and the length and width of its bounding box.
- **Lost Defect:** Inspection systems usually operate in real time, which can cause them to return inaccurate data. Thus, the characteristics of the detected individual defects can be inaccurate or an individual defect may even go undetected. These individual defects, which do exist on the surface of the inspected material but were not detected by the inspection system, are called lost defects.
- **Periodical Defect:** A set of individual defects on the same product that have been generated periodically by the same defective roll. In order to be considered a periodical defect, all individual defects within the set must fulfill the following conditions:
  – 1. All individual defects must be located in the same transversal position.
  – 2. All individual defects must have the same morphological characteristics, as they have been generated by the same flaw in the same defective roll.
  – 3. The separation between two consecutive individual defects must be constant. This separation is the period length of the periodical defect.
  Although this is the definition of an ideal periodical defect, the uncertainty in the input data must be taken into account. This means that the transversal positions, dimensions and areas of the individual defects that form a periodical defect may be slightly different. Furthermore, the distance between two consecutive individual defects may not be exactly constant and lost defects may occur. Similarly, the type assigned to these individual defects may not be the same. All stars in Figure 4 form a set which meets the above conditions, so it can be considered a periodical defect.
  - **Partial-periodical Defect:** A set that meets the conditions of periodical defect but is formed by a low number of individual defects is called a partial-periodical defect. Sometimes an object attached to a roll that is generating defects periodically can come off by itself. In these cases, the number of defects generated may be small. These cases are not of our interest, since the roll is not defective. For this reason, periodical defects with a low number of individual defects should not be considered as such and, therefore, should not be detected. This is the case of the individual defects represented by squares in Figure 4.

### 3.1. Solution Representation

Usually, inspection systems provide a large amount of information about each individual defect. In the proposed approach, the only information needed about each individual defect is its position (defined by its transversal and longitudinal coordinates), its area (in pixels), its dimensions (length and width) and its type (hole, scratch, roll mark, *etc.*). Individual defects will be represented by a tuple whose members correspond to each one of the characteristics mentioned above. Each one will be represented using one of these tuples.

(1) id={tr,lo,ar,le,wi,ty}

where *tr* and *lo* are the transversal and longitudinal coordinates of the individual defect, *ar* represents its area, and *le*, *wi* and *ty* its length, width and type respectively.

Since the aim of this study is the detection of periodical defects, an abstraction to represent them is also necessary. As periodical defects are composed by a set of individual defects generated by a defective roll periodically, they can be considered as a periodical pattern. This pattern can be defined by the following features.

(2) pd={n,u,h,p,t,a,l,w,s}

where *n* is the number of individual defects that belong to the periodical defect, *u* is the number of undetected individual defects, *h* is the highest number of consecutive undetected individual defects, *p* is its period length, *t* is its transversal position and *a*, *l* and *w* are the average area (in pixels), length and width of the individual defects that belong to the periodical defect. The feature *s* represents a set that stores the individual defects that make up the periodical defect (feature *n* is the cardinal of this set). Each periodical defect is characterized using one of these tuples.

In the remainder of the paper, the characteristic *X* of an individual defect is referred to by *id.X* and the feature *Y* of a periodical defect is referred to by *pd.Y*. The *i^th^* element of the set of a periodical defect is denoted by *pd.s.id_i_*. All individual defects within the set are ordered by their longitudinal coordinate. Thus, the individual defect within a periodical defect whose longitudinal coordinate is lowest is denoted by *pd.s.id*_1_, and the individual defect whose longitudinal coordinate is greatest is denoted by *pd.s.id_pd.n_*. Thereby, every pair of elements within the set of a periodical defect must meet Equation (3).

(3) $$\begin{array}{cc} pd.s.id_{i}.lo>pd.s.id_{j}.lo & \forall i,j\;i>j \end{array}$$

As mentioned at the beginning of this section, the characteristics of individual defects have been taken with uncertainty. For this reason, all the individual defects that make up a periodical defect will not be located in the same transversal position, due to this uncertainty (although the transversal coordinates will not be equal, they will be very similar). The same situation occurs with the area, width and length. Thus, features *t*, *a*, *l* and *w* shall contain the average value of the characteristics *tr*, *ar*, *le* and *wi* of the individual defects that integrate the periodical defect, as defined in Equations (4–7) respectively.

(4) $$pd.t=\frac{1}{pd.n}\sum_{i=1}^{pd.n}pd.s.id_{i}.tr$$

(5) $$pd.a=\frac{1}{pd.n}\sum_{i=1}^{pd.n}pd.s.id_{i}.ar$$

(6) $$pd.l=\frac{1}{pd.n}\sum_{i=1}^{pd.n}pd.s.id_{i}.le$$

(7) $$pd.w=\frac{1}{pd.n}\sum_{i=1}^{pd.n}pd.s.id_{i}.wi$$

The value of the feature *p* contains the average distance between two consecutive individual defects belonging to the periodical defect (taking into account the undetected individual defects).

(8) $$pd.p=\frac{pd.s.{id}_{pd.n}-pd.s.id_{1}}{pd.n+pd.u-1}$$

Finally, the value of the feature *h* (the highest number of consecutive undetected individual defects) can be calculated as shown in Equation (9).

(9) $$pd.h=\frac{\underset{1\leq i\leq pd.n-1}{\max}(pd.s.id_{i+1}.lo-pd.s.id_{i}.lo)}{pd.p}-1$$

This is the way in which each periodical defect is represented. The output provided by the clustering method used by the proposed sensor system consists of a collection of these tuples.

### 3.2. Detection of Periodical Defects

In this section, the way that periodical defects are detected is discussed. Initially, Section 3.2.1 shows how the initial values of a periodical defect are established. The initial solution is completed by adding individual defects that meet the conditions of a periodical defect using a clustering algorithm described in Section 3.2.2.

#### 3.2.1. Initial Solution

**Figure 5.**
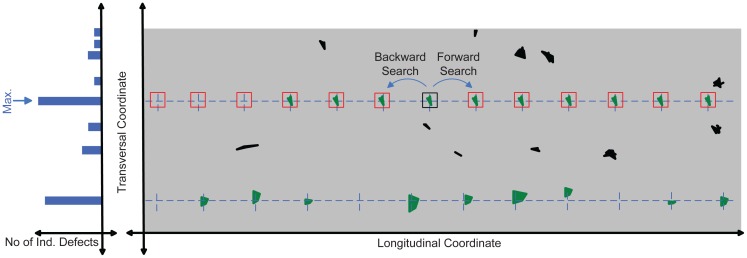
Clustering algorithm.

The amount of time available for detection may be limited. Thus, the search should examine the transversal coordinates most likely to have periodical defects first (the position of an individual defect is defined by its longitudinal and transversal coordinates, but the position of a periodical defect is defined by its transversal position only). To do this, a histogram representing the number of individual defects located at each transversal position is calculated, as shown in Figure 5. The transversal position containing the maximum of the histogram is chosen to start the search. In order to start a search at that transversal position, a periodical defect is created. Its tuple is initialized including an individual defect located at the transversal position in which periodical defects are being sought (any of them). In addition, before starting the search, the theoretical period length (*tpl*) of the periodical defects to be detected should be established (the theoretical period length of a roll could be defined as the period of the periodical defects generated by it). Generally, the theoretical period length of a roll is its perimeter, but this depends on the production process of each material. If multiple rolls are used in the production process, a search should be performed for each one of them (using their theoretical period lengths). Thus, the initial values of the features of the periodical defect are those shown in Equation (10).

(10) $$\text{pd}=\{1,0,0,\text{tpl},\text{s}.\text{i}d_{1}.tr,s.id_{1}.ar,s.id_{1}.le,s.id_{1}.wi,s\}$$

#### 3.2.2. Clustering Algorithm

Once the transversal position, in which a periodical defect is going to be sought, has been selected and an initial solution (a periodical defect with only one individual defect included in its set and located at that transversal position) has been created, applying a clustering algorithm, other individual defects are added to the set. The clustering algorithm aims to find individual defects that match the one included in the initial solution and include them in the set, with the objective of obtaining the features of the periodical defect located in the transversal position determined by the histogram (in the case where a periodical defect actually exists there). These individual defects are those that meet the conditions of being a periodical defect, explained above. To perform this task, the theoretical period of each roll involved in the production process should be taken into account.

**Figure 6.**
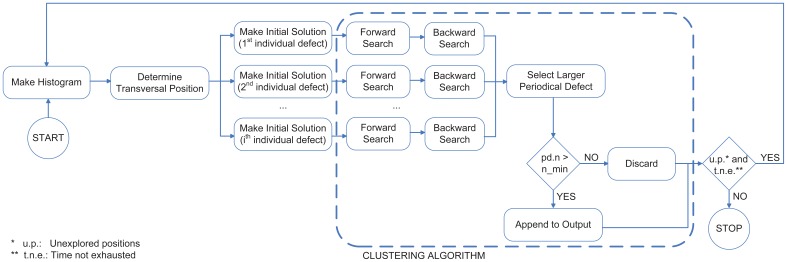
Steps of the clustering method.

Figure 6 shows the main steps of the clustering method. Accordingly, once the initial solution has been created and the clustering algorithm has been executed, periodical defects placed at other transversal positions are sought. To do this, the histogram is calculated again, without taking into account the individual defects included in a periodical defect previously detected. An initial solution is created for that transversal position and the clustering method is applied again. These steps are repeated until all transversal positions have been checked or the time available for performing the detection is exhausted.

In Figure 5 an example with two periodical defects is shown. One of them, shown at the top of the figure, is located at the transversal position in which the histogram has its maximum. The initial solution must contain an individual defect located in that transversal position (in the example, the individual defect that is within a black square). The clustering algorithm should look for individual defects that match it. These must be located in the same transversal position; they must be characterized as the same type and have the same area, length and width. Moreover, their longitudinal position must be consistent with the longitudinal position of the individual defect included in the initial solution, considering the theoretical period length of the rolls involved in the production process (the initial value of the feature *p* of the periodical defect). Assuming the case where the initial periodical defect is the one shown in Equation (10) and the individual defect included in the initial solution (designated as *iid* and at this point, *pd.s.id*_1_ is the one shown in Equation (11), individual defects whose position fits with it are those located in (*iid.tr, iid.lo* ± *N* · *pd.p*), where *N* is a natural number. These positions are represented in Figure 5 by dashed blue lines.

(11) iid={tr,lo,ar,le,wi,ty}

This approach works properly only in ideal situations, where all individual defects are located at the same transversal position, the longitudinal separation between them is constant, there are no lost defects, and their dimensions (length, width and area) are identical. As already remarked, input data may be inaccurate, so a real periodical defect is most similar to that shown at the bottom of Figure 5, where the individual defects may have different length, width, area, transversal position and type. In addition, their separation may not be constant (in longitudinal direction) and some individual defects may have not been detected. Thus, various tolerances should be used when determining where other individual defects of the periodical defects should be located and when deciding whether an individual defect matches the features of the periodical defect or not. Related to the position, it is necessary to establish two tolerances: one for the transversal position and another for the longitudinal position. The transversal tolerance (hereinafter t_tol) establishes the maximum allowable difference between the transversal position of an individual defect and the feature *t* of the periodical defect where it aims to be included (in *mm*). The longitudinal tolerance (hereinafter p_ratio) establishes the maximum allowable difference between the longitudinal position of an individual defect candidate to be included in a periodical defect, and the longitudinal position where it should be located. This tolerance is defined as a percentage of the *p* feature of the periodical defect. Thereby, individual defects whose position should be accepted as similar to the individual defect defined in Equation (11), taking into account these tolerances, are those located in (*iid.tr* ± *t_tol, iid.lo* ± *N* ·*pd.p* ± *pd.p* · *p_ratio*). These areas are represented in Figure 5 by red squares and they constitute the search space of the algorithm. The width and length of these squares are defined by t_tol and p_ratio respectively.

The search space is explored in two steps: the forward search and the backward search. Both start from the position of the initial defect, located in (*iid.tr, iid.lo*). When performing the forward search, the algorithm starts checking whether an individual defect is located in (*iid.tr* ± *t_tol, iid.lo* ± *pd.p* ± *pd.p* · *p_ratio*). If there is an individual defect within that area (candidate individual defect, or *cid*), its characteristics should be checked. That individual defect is only included in the periodical defect if it meets the following conditions:

(12) $$\text{cid}.ar\in [\frac{pd.a}{a\_\text{ratio}/100},pd.a\cdot a\_\text{ratio}/100]$$

(13) $$\text{cid}.le\in [\frac{pd.l}{l\_\text{ratio}/100},pd.l\cdot l\_\text{ratio}/100]$$

(14) $$\text{cid}.\text{wi}\in [\frac{\text{pd}.\text{w}}{\text{w}\_\text{ratio}/100},pd.w\cdot w\_\text{ratio}/100]$$

where *a_ratio*, *l_ratio* and *w_ratio* are the morphological tolerances. They establish the maximum allowable difference between the area, length and width of an individual defect candidate to be included in a periodical defect, and the area, length and width that it should have: *pd.a*, *pd.l* and *pd.w* respectively. These tolerances are defined as a percentage of the *a*, *l* and *w* feature of the periodical defect. In addition, before starting the execution of the clustering algorithm, individual defect types that are allowed to form the periodical defects must be established. The type of the candidate individual defect must be one of the allowed types.

If the individual defect meets these conditions, it is included in the periodical defect (if there is more than one individual defect within the area that meets the above conditions, the chosen defect is the one closest to the ideal position).

Each time a candidate individual defect is included in the periodical defect, its *n* feature is incremented by one and the features *t*, *a*, *l*, *w* and *p* are updated using Equations (4–8) respectively.

This process is repeated to check the other squares (search areas) in the search space, *i.e.*, areas centered at the points (*iid.tr, iid.lo* + *pd.p*), (*iid.tr, iid.lo* + 2 · *pd.p*), (*iid.tr, iid.lo* + 3 · *pd.p*) and so on, until the end of the web material is reached.

Note that the longitudinal positions of the points above are calculated using *pd.p* instead of the theoretical period length since it may have been estimated with low precision. However, *pd.p* contains the actual average distance between individual defects. Thus, the error in the case that the theoretical period length has not been calculated with sufficient accuracy is minimized. In order to further minimize the error in these calculations, both the transversal and the longitudinal position of these points should not be determined as a function of *iid.tr* and *iid.lo* but by the average transversal position (*pd.t*) and the longitudinal position of the last defect inserted in the set (*pd.s.id_pd.n_*). In this way the area to be checked until the end of the web material is reached is defined by Equation (15).

(15) $$\left(pd.tr\pm t\_\text{tol},pd.s.id_{pd.n}.lo+pd.p\pm pd.p\cdot p\_\text{ratio}\right)$$

Each time an individual defect is introduced into the set, it is the one whose longitudinal position is higher, *i.e.*, the latest individual defect inserted into the set is always *pd.s.id_pd.n_* during the forward search. In this way the area defined by Equation (15) changes every time an individual defect is introduced into the set, defining the area where the next one must be sought.

**Figure 7.**
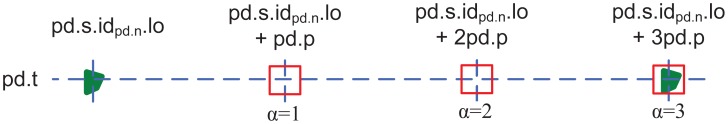
Forward search with lost defects.

If there are no defects within the area defined by Equation (15) (or none satisfies the conditions Equations (12,13,14), the feature *pd.u* is increased by one and the feature *pd.h* is updated by Equation (9). In addition, the search area must be defined taking into account this situation. To do this, an auxiliary variable *α* is defined, with a default value of 1. Each time an individual defect is inserted into the set, the value of *α* is reset to 1. Like *pd.u*, when there are no individual defects within the search area its value is incremented by one. Thus, the search can continue even when individual defects were not inserted into the set, as shown in Figure 7. The general expression for defining the search area during the forward search is shown in Equation (16).

(16) $$\left(pd.tr\pm t\_\text{tol},pd.s.id_{pd.n}.lo+\alpha pd.p\pm pd.p\cdot p\_\text{ratio}\right)$$

When many individual defects exist in the web material inspected, some of them may be located in close transversal positions and have similar morphological characteristics by chance. These defects could be incorrectly clustered as a periodical defect. To avoid this situation, it is necessary to set two thresholds, defined as follows.

- **Size Threshold (n_min):** Sets the minimum number of individual defects that a periodical defect must have to avoid being considered a partial-periodical defect.
- **Undetected Defects Threshold (max_skips):** Maximum allowable number of individual defects undetected consecutively.

By using size thresholds, the probability that a set of defects, such as those mentioned above, are classified as a periodical defect is minimized. Also, it establishes the difference between partial-periodical and periodical defect. In addition, the undetected defects threshold can be used by technicians to set the highest number of lost defects they deem acceptable (that is, the maximum allowed value for the parameter *h* of a periodical defect and the highest value that can take the variable *α*). If the technician considers it unnecessary to stop the production line when the individual defects that form the periodical defects are subtle, a small value for *h* can be established (subtle defects are more difficult to detect, and therefore many of them may go undetected). On the contrary, if it is considered necessary to stop the line whenever periodical defects are being generated (even though they are very subtle) a large value for *h* can be established. During both searches, forward and backward, Equation (17) must be fulfilled:

(17) α<max_skips+1

During the forward search, when the end of the web material is reached or Equation (17) ceases to be met, it ends and the backward search begins. The backward search looks for individual defects to be included into the set from the position where the first individual defect is located (*pd.s.id*_1_.*tr, pd.s.id*_1_.*lo*) to the beginning of the web material. The manner in which individual defects are sought is analogous to the forward search. The area within which the next individual defect is sought is defined by an expression similar to that used during the forward search, but in this case it must be taken into account that the last defect included in the set is the one that is located in a lower longitudinal position, *i.e.*, *pd.s.id*_1_. Whenever an individual defect is included in the set, it will become the new *pd.s.id*_1_ (since its longitudinal position is the lowest). Thus, the search area for the backward search is defined by Equation (18).

(18) $$\left(pd.tr\pm t\_\text{tol},pd.s.id_{1}.lo-\alpha pd.p\pm pd.p\cdot p\_\text{ratio}\right)$$

When the beginning of the web material is reached or Equation (17) ceases to be met, the backward search stops and the whole search ends.

**Figure 8.**
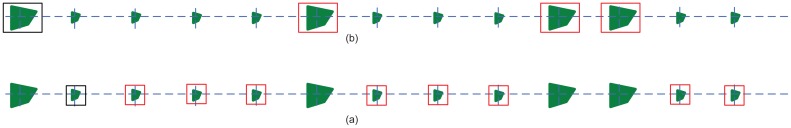
Differences in detection depending on the initial solution.

A very important aspect that must be taken into account is that the individual defect selected to form the initial solution greatly affects the search, since the values of their characteristics *iid.tr*, *iid.ar*, *iid.wi* y *iid.le* are assigned to features *pd.t*, *pd.a*, *pd.w* y *pd.l* of its periodical defect respectively at the beginning of the search. Thus, the clustering algorithm includes in the periodical defect only those individual defects whose characteristics are similar to those of the individual defect included in the initial solution. Therefore, if this individual defect was detected by the vision system incorrectly (e.g., assigning a value to its dimensions and area much larger than they should be) the clustering algorithm will not work properly, since it will include only individual defects similar to it (whose dimensions and area are also incorrect), as shown in Figure 8. In this figure, the individual defect chosen to form the initial solution is surrounded by a black square, and individual defects included in the periodical defect by the clustering algorithm are surrounded by red squares. The clustering is better in the cases where the individual defect included in the initial solution is more like the majority of individual defects that form the periodical defect. Since the individual defect most appropriate to form the initial solution can not be determined before running the algorithm, it must receive as many initial solutions as individual defects are located at the transversal position determined by the histogram (see Figure 6). For each of them, both forward and backward searches are applied. The largest periodical defect (*i.e.*, the one whose feature *n* is greater) is the one chosen to be part of the output, but only if its feature *n* is greater than the size threshold.

### 3.3. Parameter Settings

The tolerances and thresholds constitute the configuration parameters of the clustering algorithm, adjusting its behavior. Thus, their values must be set appropriately taking into account the type of web material inspected and the inspection system used. If the uncertainty introduced by the inspection system is small, the parameters should be restrictive, and if it is large, the parameters should be more permissive.

For each case in which the clustering method is going to be used, the optimal value of each parameter must be set. To do this, the output provided by the sensor must be assessed. Thus, the parameter configuration whose output has the best assessment can be considered the best. A method to perform this assessment using discrepancy based metrics is proposed in Bulnes *et al.* [11]. In order to determine the optimal parameter values in this way, it is necessary to have several examples of the web material for which the proposed sensor is going to be used, where the inspection task is carried out by the same system that provides the input data. For each one of these examples, an expert technician must make the detection of periodical defects manually a priori. This detection will be considered perfect and used as reference. The assessment method accounts for the differences between the output provided by the proposed sensor and the reference, and returns a metric value within the closed interval [0, 1] (the higher the value, the fewer differences). Specifically, the F-Measure metric was chosen for this purpose [29]. The selected parameter configuration is the one that provides a higher average metric value.

## 4. Practical Application: Detection of Periodical Defects in Hot Steel Strips

A practical application has been taken into consideration to assess the functioning of the sensor presented in this paper: hot steel strips. It has been chosen because it is a case in which periodical defects are difficult to detect. This is because there are several rolls involved in the production process, the number of surface defects is usually high and the input data are very noisy.

### 4.1. Industrial Context

**Figure 9.**
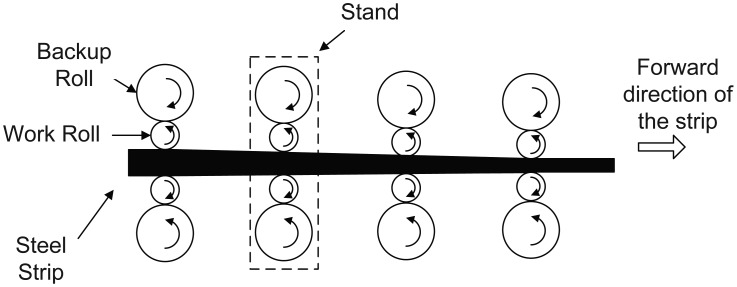
Steel strip at the finishing mill.

In this application, the generation of periodical defects occurs in the finishing mill (Figure 9), where hot steel is stretched and flattened by several rolls, exerting great pressure on steel until it reaches the form of a strip of great length. During the rolling process, the strip has to pass through seven stands. Each one consists of four rolls: two backup rolls and two work rolls. The backup rolls transmit force to the work rolls, which are those in direct contact with the steel. For this reason, the work rolls are those that can generate periodical defects on the surface of the strips. The surface of the work rolls must be completely smooth to obtain a perfect rolling. Besides the typical problems on rolls (cracks or attached objects), in this particular case they become worn very quickly [30], which makes the inspection even more important so as to replace the malfunctioning rolls as early as possible.

**Figure 10.**
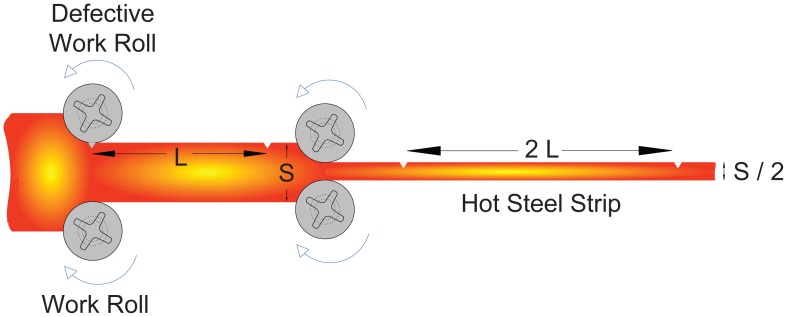
Relation between the reduction of thickness and the elongation of the strip.

As discussed in Section 3.2.1, the theoretical period length of the rolls involved in the production process must be known before starting the detection. Thus, the time required to perform the detection can be reduced by searching only periodical defects with such periodicities, instead of iterating through a range of possible values as made by Traxler *et al.* [25]. In our practical application, the theoretical period length does not correspond to the perimeter of the rolls since the length of the material varies, but it can be calculated easily. The forces applied during rolling produce friction on the strip, which is lower in the transversal direction than in the forward direction of the strip, cause the stretching of the strip in a forward direction and not sideways. Figure 10 shows an example in which a defective roll generates a periodical defect whose period length is initially *L* (the perimeter of that roll) when the thickness of the strip is *S*. Passing through the next stand, the thickness of the strip is reduced to *S/*2. Since the volume of the steel remains constant, the length of the steel increases proportionally, causing the separation between two consecutive individual defects within the periodical defect to increase to 2*L*. Considering the separation between each pair of work rolls of the same stand and their radii, the theoretical period of each cylinder can be calculated by Equation (19) [31].

(19) $$p_{i}=(2\pi )r_{i}\prod_{j=i+1}^{n}\frac{1}{1-b_{j}}$$

where *n* is the number of stands in the finishing mill, *p_i_* is the theoretical period length of the work rolls of the *i^th^* stand and *r_i_* is its radius. The reduction factor *b_j_* measures the thickness difference of the strip due to passing through the *j^th^* stand.

(20) $$b_{j}=\frac{h_{j-1}-hj}{h_{j-1}}$$

where *h_k_* is the thickness of the steel strip after passing through the *k^th^* stand.

### 4.2. Experimentation

To perform the experimental phase, 36 strips produced in the ArcelorMittal Avilés factory (Spain) have been used. In order to achieve representative results, strips with different characteristics have been chosen. The number of periodical defects in each strip varies from 1 to 5, where the number of individual defects that form them varies from 20 to 250 and the number of individual defects in each strip varies from 80 to nearly 5000. They are also strips of different types of steel, different thicknesses and dimensions.

All these strips have been inspected during their production process using an inspection system by Parsytec [32] which is able to both detect and classify individual defects and also perform the detection of periodical defects. This system uses CCD cameras that take pictures in grayscale with a resolution of 768 × 494. Those images are overlapped to ensure complete coverage. Depending on the side and the camera, the overlap varies from 5% to 15%. The separation between the cameras and the strip is 2 meters on the top side and 3.67 meters on the bottom side. Since the steel strips are moving very fast (between 11 and 17 meters per second) exposure time of the cameras is low: 100*μs*. The strips are illuminated by xenon strobes emitting 120 flashes per second (12*μs* of duration each). The detection of periodical defects by the detection method proposed has been entirely written in *C*++ using *Microsoft Visual Studio 2008*. These experiments were performed on a computer that uses an *Intel Core2 Quad Processor Q9400*, taking 7 seconds (on average) to analyze a full strip.

The data provided by the inspection system about the detection and classification of individual defects is used as input data, while the periodical defect detection is used for comparison with the output provided by the proposed sensor, in order to check whether it represents an improvement with respect to an important system used worldwide.

The first step in applying the proposed method is to determine the optimal parameter configuration of the clustering method for this case using that inspection system. Then, this configuration is used to perform the testing phase. In order to avoid obtaining an overfitted configuration, the strips selected for experimentation have been divided into two different groups of equal size (18 strips each). The first one is used to obtain the optimal parameter configuration and the second one to perform the testing.

Using a full-factorial experimental design executed by a grid composed of 60 computers, the optimal parameter configuration was obtained. The one that provides better average results for the first set of strips is shown in Table 1.

The values obtained for the tolerances applied to the morphological characteristics of individual defects are very high in the optimal configuration due to the low accuracy under which they were obtained in this particular case. This means that two different individual defects are considered morphologically similar if the area, length and width of one of them is 100 times greater than the other. Practically this means that the morphological characteristics of the individual defects are not taken into account in this case, where the detection of periodical defects are performed based on the position and type of the individual defects only. Moreover, the value of the parameter *max_skips* is also very large because the occurrence of lost defects is very common.

The last step of the experimentation is to make the detection of periodical defects for the second set of strips using the proposed sensor, applying the obtained parameter configuration for the clustering method. The results are compared with those provided by the Parsytec system in order to assess whether the behavior of the proposed sensor is good enough to work in a real industrial environment.

### 4.3. Results

**Figure 11.**
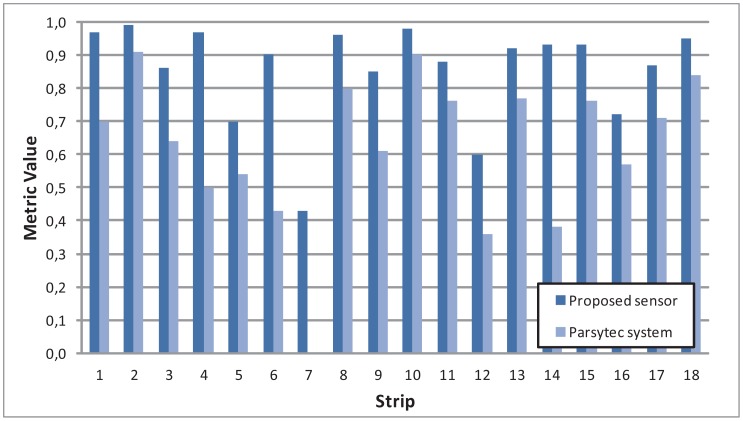
Comparison between the proposed sensor and the Parsytec system.

The obtained results after using the proposed sensor on the set of test strips is shown in Figure 11 with the results provided by the Parsytec system for that same set. The numerical data are also listed in Table 2. These results show a clear improvement, since the output provided by the proposed sensor has obtained a better rating than the Parsytec system for all strips. Table 3 shows the average metric values provided by each system.

These results suggest that the quality of the output of the proposed sensor is better than that provided by the Parsytec system. To verify this assertion, a statistical test needs to be applied. The first step is to check whether the data are normally distributed. To do this we used the Shapiro–Wilk test (All statistical calculations were performed using R), concluding that the hypothesis of normality for the data provided by the proposed method can be rejected (*p*-value = 0.0011), but not for data provided by the Parsytec system (*p*-value = 0.1222). Due to the lack of normality, a one-sided Wilcoxon test for paired data should be used. The distribution *D* is defined as the difference between the metric value provided by the proposed method and system Parsytec for each strip. Thus, the hypotheses to be tested using this method are defined as:

$$\{\begin{array}{l} H_{0}:\text{the median of the distribution D is less than or equal to}\;0\\ H_{1}:\text{the median of the distribution D is greater than}\;0 \end{array}$$

Applying the Wilcoxon test, a *p*-value of 0.00011 is obtained, allowing the rejection of the null hypothesis with confidence. Thus, it can be concluded that the results obtained by the proposed sensor are statistically superior to those provided by the Parsytec system.

## 5. Conclusions

This paper has presented a sensor system based on computer vision capable of detecting periodical defects in web materials during their production. This kind of defects requires an early detection, as the defects can greatly degrade the quality of the produced material. Therefore, the clustering method used by the sensor is fast and enough to be able to provide the results within a few seconds without using a powerful computer.

All steps of the clustering method have been detailed. It is based on the position and the morphology of the surface defects to determine whether a set of them may have been generated periodically by a defective roll. In order to function properly even with noisy input data, a tolerance is applied to each of the defect characteristics that are taken into account. Thus, by changing the value of each tolerance, the behavior of the proposed method can be adapted to operate optimally for the web material to be inspected.

To verify the correct operation of the sensor system, it was applied to the detection of periodical defects in hot steel strips. The optimal configuration for this particular case was determined and then used in real steel strips. The results were compared with those provided by an inspection system that is widely used for the detection of periodical defects, which is considered the state-of-the-art in this field. The comparison proves the success of the proposed sensor and its capacity to detect periodical defects in any industry.

## Figures and Tables

**Table 1.** Optimal configuration.

**Parameter**	**Value**
n_min	18
max_skips	20
a_ratio	100
w_ratio	100
l_ratio	100
t_tol	56
p_ratio	7

**Table 2.** Metric values obtained for each strip.

**Strip**	**1**	**2**	**3**	**4**	**5**	**6**	**7**	**8**	**9**	**10**	**11**	**12**	**13**	**14**	**15**	**16**	**17**	**18**
Proposed sensor	0.97	0.99	0.86	0.97	0.70	0.90	0.43	0.96	0.85	0.98	0.88	0.60	0.92	0.93	0.93	0.72	0.87	0.95
Parsytec system	0.70	0.91	0.64	0.50	0.54	0.43	0.00	0.80	0.61	0.90	0.76	0.36	0.77	0.38	0.76	0.57	0.71	0.84

**Table 3.** Average F-Measure metric values.

	**Average Metric Value**
Proposed method	0.86
Parsytec system	0.62
